# Analysis of the Contribution of the Road Traffic Industry to the PM2.5 Emission for Different Land-Use Types

**DOI:** 10.1155/2014/821973

**Published:** 2014-11-04

**Authors:** Peng Xu, Wei Wang, Jiawei Ji, Shunyu Yao

**Affiliations:** Hohai University, Nanjing 210024, China

## Abstract

Road dust and vehicle exhaust are the main sources of air pollution in cities, especially in recent years with the quantity of vehicles and transportation construction continuously soaring; the hazy weather has been a dominant urban pollution form which is widely concerned by the Chinese society. By establishing a relationship model between traffic and land use, then applying analytic hierarchy process on the data from air quality monitoring station, this paper concludes the influence of different traffic behavior on air pollution which provides support to abate urban air pollution caused by traffic reasons through taking measures to control traffic.

## 1. Introduction

In recent years, the urban air quality problem got widely social attention. At present, the particulate pollution has become a primary factor affecting China's urban air quality [1]. Road dust and motor vehicle exhaust are the main sources associated with transport industry among large number of pollution sources (road dust, construction fugitive dust, bunker coal, motor vehicle exhaust, biomass burning, etc.) [2]. Their pollution contribution is always greater than 50% [3]. According to the results of previous research, road dust is the main source of PM10 in urban atmosphere and motor vehicle exhaust mainly affects the concentration of PM2.5 and nitrogen oxides [4].

As the problem of air pollution is growing significant and people care more about their own survival environment, the control of the particulate matter pollution has been the focus of prevention and control of atmospheric pollution. Particulate pollution from traffic factor will continue to rise with the rapid increase of the amount of vehicles urban road construction. This paper tries to analyze traffic characteristics and the influence under different forms of urban land and then set up related model to serve as a reference for the urban pollution control.

## 2. Model Establishment and Illustration

Urban land is often associated with economic system, social system, and traffic system together as a scarce resource. In terms of traffic system, different forms of land use determine not only the trip generation and trip attraction, that is to say, the distribution form of transportation, but also the traffic structure to a certain extent [5]. Traffic form is different due to the properties of urban land, so the influence on urban air quality is different. Traffic form refers to a common concept such as traffic volume, trip generation and trip attraction, and vehicle features. In the city within the scope of a certain land, different traffic form shows the different volume, distribution, diffusion, and so on.

The urban land types can be divided into 10 categories according to China's* Urban Construction Land Classification and Planning Standards *for GB50137-2011, respectively, residential land, land for public management and public service, land for industry, land logistics warehousing, land for business services facilities, land for roads and traffic facilities, and land for public facilities green space and square waters and other sites. Different land uses correspond to different traffic demand [6]; that is to say, generation trips, traffic conditions, and vehicle characteristics in 10 different lands vary per unit area. Generally, the all-weather traffic volume is too heavy in commercial land which is always in the city center, so exhaust emission is relatively too much. And in residential land, traffic is periodic; traffic volume is larger in the morning and evening rush hour. The traffic volume is small at the rest time; the air quality is good. And air quality is poor in the industrial land and the land for storage because of big proportion of freight traffic. On the other hand, traffic construction on the outskirts is more than the old, so traffic dust is relatively serious to the older sections. Again on the other hand, air flow dissipation effect is much less than the suburbs with the older sections' buildup.

**Figure 1:**
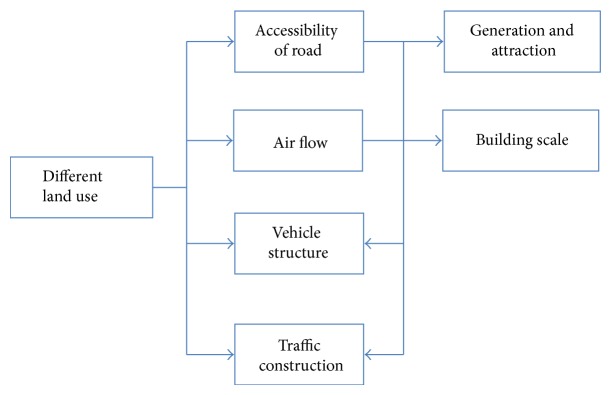
Mutual influence between traffic factors affecting air condition.

In conclusion, the traffic factors affecting air quality can be mainly divided into four categories: accessibility of road, vehicle structure features, air flow, and traffic construction scale. Mutual influence relations are shown in Figure 1.

**Figure 2:**
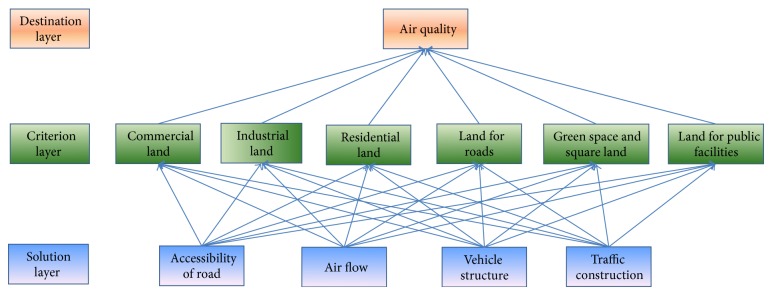
Hierarchical chart.

This paper compared and analyzed the four different traffic factors affecting air quality combined with pollutant concentration data of known cities by AHP method. The AHP is a combination of qualitative and quantitative, systematic and hierarchical method which is effective and practical in dealing with complex decision problem. This paper selects six lands for modeling analysis including residential land, commercial land, land for roads and traffic facilities, green space and square land, land for public facilities, and land for industry. Thus this section established the model of hierarchical structure between urban land and the air quality, shown in Figure 2.

### 2.1. Introduction to the Modeling

The model was established based on analytic hierarchy process (AHP) in this chapter for the analysis of weight of traffic factors consists of accessibility of road, air flow, vehicle structure, and traffic construction scale impact on air quality, soluted by yaahp software. So air quality is in the destination layer; criterion layer concludes concentration of PM2.5 of different land and the solution layer concludes the four traffic factors. Hierarchy chart as shown in Figure 2 finalizes weights for different traffic factors impact on urban air quality by PM2.5 concentrations in different land use.

The data of PM2.5 concentrations tested by air monitoring stations are comprehensively influenced by various factors, so it needs to get rid of the influence of other factors before calculation. According to previous study, mass concentration of PM2.5 is 50%–80% of PM10 in Beijing and Guangzhou. In general, the contribution rate of dust for PM10 accounted for 20%~60% and the motor vehicle emissions for about 5%~20% [7]. Thus, it can estimate the contribution rate of transportation factors of PM2.5.

### 2.2. Hypotheses of the Modeling

(1) The yaahp software applies a 1~9 scale on behalf of the importance of every two indicators. The model analyzes four traffic factors which influence degree of six land uses, that is, accessibility of road, vehicle structure features, air flow, and traffic construction scale. Because the weight which is taken into account is subjective, the judgment below is based on expert's experience:

 on commercial land, accessibility of road compared to air flow has the same importance (1);
 comparing accessibility of road to vehicle structure features, the former is more important than the latter (5);
 comparing accessibility of road to traffic construction scale, the former is tinily more important than the latter (2);
 comparing air flow to vehicle structure features, the former is more important than the latter (4);
 comparing air flow to traffic construction scale, the former is slightly more important than the latter (3);
 comparing vehicle structure features to traffic construction scale, the latter is tinily more important than the former (1/2).

Judgment matrix of traffic factors on commercial land is shown in Table 1. The other lands are similar to commercial land, shown in Tables 2, 3, 4, 5, and 6. *W*_*i*_ is the weight that traffic factors impact on air quality with. 

CR is consistency ratio which is more tending to zero; the model has the better consistency. The CR < 0.1; here, it means the sort has relatively satisfactory consistency.

(2) The concentration data used in the model is the results of stripping out traffic factors. So when calculating standard percentage, standard concentrations are the product of primary index concentration and average contribution rate; here average contribution rate is 40%.

(3) The pollution index uses a 1~9 scale as the yaahp software uses a 1~9 scale. *M* is excessive percentage. When *M* ⩽ 0, *K* = 1; when 0 < *M* ⩽ 25, *K* = 2; when 25 < *M* ⩽ 75, *K* = 3; when 100 < *M* ⩽ 150, *K* = 4; when 75 < *M* ⩽ 100, *K* = 5; when 100 < *M* ⩽ 150, *K* = 6; when 150 < *M* ⩽ 200, *K* = 7; when 200 < *M* ⩽ 250, *K* = 8; when *M* > 250, *K* = 9.

## 3. Analysis of Cases

This section takes Nanjing as a case. Nanjing located in the midlatitude eastern China belongs to north subtropical monsoon climate zone, and the wind conversed apparently between summer and winter. In addition, Nanjing has economic industry developed in Jiangsu Province and the Yangtze River Delta and is a typical city of our southern country. It can well reflect the east China area general urban air pollution influence degree.

### 3.1. Data Explanation

The model's data comes from Nanjing Municipal Environmental Protection Bureau's official website.  Table 7 is the average concentration of PM2.5 in the fourth quarter of 2013 in Nanjing by randomly sampling the statistics and has the use value. There are 10 control points existing in Nanjing, Olympic center, ZhongHuamen, MaiGaoqiao, RuiJin Road, ShanXi Road, and XuanWu River which, respectively, represent the urban land for public facilities, land for roads, industrial land, residential land, commercial land, and green land.

1. Table 8 is the concentration of PM2.5 only from traffic factors [5] based on Table 7.
2. Table 9 is the excessive percentage of 24-hour average levels of PM2.5 based on Table 8. The first grade indexes concentration of PM2.5 is 35 *μ*g/m^3^ per day, stripping out the traffic factors; take 14 *μ*g/m^3^ per day.
3. Table 10 includes pollution level *K* of pollutants on the basis of Table 9. Because of the yaahp using a 1~9 scale, the pollution index of the pollution level of *K* also uses a 1~9 scale.

### 3.2. Model Solution

(1) First compare the air quality between different urban lands; it is concluded that urban land pollution is affected by the traffic factor; the greater the numerical says, the more serious the pollution is, as shown in Table 11.

(2) Sort out the weights of traffic factors corresponding to each land use into Table 12, and the weights of four different traffic factors on the overall impact of PM2.5 are got. The greater the value is, the greater the extent of the pollution to the air is.

## 4. Result Analysis

From Table 9, it is obvious that the concentrations of PM2.5 in Nanjing content exceed bid badly, and traffic factors related to motor vehicle are the main source. In addition, because the diameter of the PM2.5 is smaller, it is more easy to enter the body's blood circulation and the harm to human body health is larger than PM10 [8]. According to data released by the Beijing Environmental Protection Bureau in 2012, PM2.5 pollution cases of motor vehicles accounted for 22.2%. And the PM2.5 pollution contribution of motor vehicle exhaust and road dust only grows with building largely and the rapid increase of the amount of vehicles in Nanjing in nearly three years.

**Figure 3:**
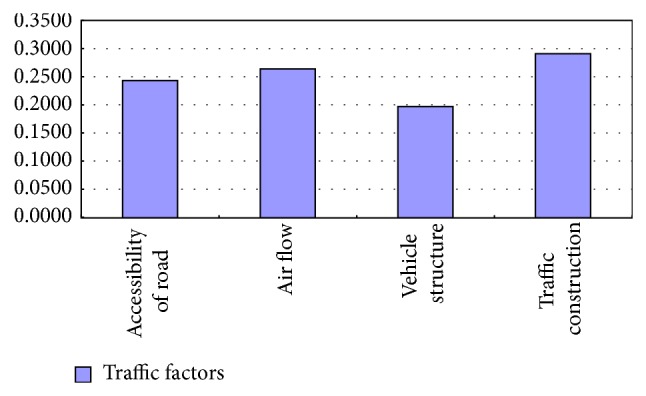
Traffic factors weights on PM2.5.

Make histogram of different traffic factors weights according to Table 12, as shown in Figure 3.

According to Figure 3, four kinds of traffic factors have a certain degree of influence on the concentration of PM2.5. Among them, the proportion of traffic construction scale is relatively large, and the accessibility of road and air flow have the similar influence.

The impact of traffic factors on commercial land is mainly the excessive exhaust emissions caused by vehicle jam and slow going. And commercial land air liquidity is poorer, appropriate to reduce traffic and improve the average speed to solve the problem of pollution. The impact of traffic factors on industrial land is mainly the excess emissions of large vehicle; large vehicle emission test should be taken to improve this problem.

## 5. Conclusion

1. The impact of traffic construction scale contributes a lot to the conclusion among the influences of four different traffic factors on the emission density of PM2.5.
2. Road dust and vehicle exhaust are the main sources of air pollution by particulate matter, and good results can be envisioned if curbing urban air pollution through governing these two factors.
3. It will be more effective to reduce air pollution by taking different measures in traffic control according to different land use purposes.
4. The data used in this paper's modeling are from a typical city's air quality monitoring result in a certain season. In fact air quality in winter is worse than in summer [9, 10]. In addition, the data comes from random air quality results in a quarter of a year. There is some deviation from actual situation, but the general status is roughly the same.

## Figures and Tables

**Table 1:** Judgment matrix of traffic factors on commercial land.

	Accessibility of road	Air flow	Vehicle structure	Traffic construction	*W*_*i*_
Accessibility of road	1	1	5	2	0.3707
Air flow	1	1	4	3	0.3880
Vehicle structure	1/5	1/4	1	1/2	0.0829
Traffic construction	1/2	1/3	2	1	0.1584

CR = 0.0101.

**Table 2:** Judgment matrix of traffic factors on industrial land.

	Accessibility of road	Air flow	Vehicle structure	Traffic construction	*W*_*i*_
Accessibility of road	1	1/2	1/2	2	0.1895
Air flow	2	1	1	2	0.3187
Vehicle structure	2	1	1	4	0.3790
Traffic construction	1/2	1/2	1/4	1	0.1127

CR = 0.0226.

**Table 3:** Judgment matrix of traffic factors on residential land.

	Accessibility of road	Air flow	Vehicle structure	Traffic construction	*W*_*i*_
Accessibility of road	1	2	1	2	0.3333
Air flow	1/2	1	1/2	1	0.1667
Vehicle structure	1	2	1	2	0.3333
Traffic construction	1/2	1	1/2	1	0.1667

CR = 0.

**Table 4:** Judgment matrix of traffic factors on land for roads.

	Accessibility of road	Air flow	Vehicle structure	Traffic construction	*W*_*i*_
Accessibility of road	1	1	1	3/4	0.2308
Air flow	1	1	1	3/4	0.2308
Vehicle structure	1	1	1	3/4	0.2308
Traffic construction	4/3	4/3	4/3	1	0.3077

CR = 0.

**Table 5:** Judgment matrix of traffic factors on green land.

	Accessibility of road	Air flow	Vehicle structure	Traffic construction	*W*_*i*_
Accessibility of road	1	1	3	1/3	0.2309
Air flow	1	1	4	1/3	0.2191
Vehicle structure	1/3	1/4	1	1/4	0.0775
Traffic construction	3	3	4	1	0.4995

CR = 0.0462.

**Table 6:** Judgment matrix of traffic factors on land for public facilities.

	Accessibility of road	Air flow	Vehicle structure	Traffic construction	*W*_*i*_
Accessibility of road	1	1	5	2	0.3707
Air flow	1	1	4	3	0.3880
Vehicle structure	1/5	1/4	1	1/2	0.0829
Traffic construction	1/2	1/3	2	1	0.1584

CR = 0.0327.

**Table 7:** The average concentration of PM2.5 of different monitoring station μg/m^3^.

	ShanXi Road	MaiGaoqiao	RuiJin Road	ZhongHuamen	XuanWu River	Olympic center
Concentration	67	76	70	75	74	84

**Table 8:** The concentration of PM2.5 only from traffic factors *μ*g/m^3^.

	ShanXi Road	MaiGaoqiao	RuiJin Road	ZhongHuamen	XuanWu River	Olympic center
Concentration	43.55	38	24.5	45	29.6	33.6

**Table 9:** Excessive percentage of pollutants.

	ShanXi Road	MaiGaoqiao	RuiJin Road	ZhongHuamen	XuanWu River	Olympic center
Excessive percentage	311.07%	271.43%	175.00%	321.43%	211.43%	240.00%

**Table 10:** Pollution level *K* of pollutants.

	ShanXi Road	MaiGaoqiao	RuiJin Road	ZhongHuamen	XuanWu River	Olympic center
Pollution level	9	9	7	9	8	8

**Table 11:** The pollution proportion of urban land.

	Commercial land	Industrial land	Residential land	Land for roads	Green land	Land for public facilities	*W*_*i*_
Commercial land	1	1	9/7	1	9/8	9/8	0.18
Industrial land	1	1	9/7	1	9/8	9/8	0.18
Residential land	7/9	7/9	1	7/9	7/8	7/8	0.14
Land for roads	1	1	9/7	1	9/8	9/8	0.18
Green land	8/9	8/9	8/7	8/9	1	1	0.16
Land for public facilities	8/9	8/9	8/7	8/9	1	1	0.16

**Table 12:** PM2.5 weight impact between traffic factors and land use.

	*P*						Total ordering
	Commercial land	Industrial land	Residential land	Land for roads	Green land	Land for public facilities	
Accessibility of road	0.3707	0.1895	0.3333	0.2309	0.1420	0.2308	0.2444
Air flow	0.3880	0.3187	0.1667	0.2191	0.2388	0.2308	0.2653
Vehicle structure	0.0829	0.3790	0.3333	0.0775	0.0907	0.2308	0.1983
Traffic construction	0.1584	0.1127	0.1667	0.4995	0.5285	0.3077	0.2920
